# Efficacy of short-course colchicine treatment in hospitalized patients with moderate to severe COVID-19 pneumonia and hyperinflammation: a randomized clinical trial

**DOI:** 10.1038/s41598-022-13424-6

**Published:** 2022-06-02

**Authors:** Alberto Cecconi, Pablo Martinez-Vives, Alberto Vera, Cristina Lavilla Olleros, Ana Barrios, Eva Fonseca Aizpuru, Pilar Roquero, Susana Hernandez Muñiz, Maria Jose Olivera, Marianela Ciudad, Ruben Pampin Sanchez, Rosa Fernandez-Madera Martínez, Azucena Bautista-Hernández, Elena García Castillo, Gorane Iturricastillo, Elena Ávalos, Diana Prada Cotado, Alvaro Alejandre de Oña, Eduardo Fernandez Carracedo, Ana Marcos-Jimenez, Ancor Sanz-Garcia, Aranzazu Alfranca, Maurizio Cecconi, Hortensia de La Fuente, Maria Angeles Sanz de Benito, Paloma Caballero, Francisco Sanchez-Madrid, Julio Ancochea, Carmen Suarez, Luis Jesus Jimenez-Borreguero, Fernando Alfonso

**Affiliations:** 1 Cardiology Department, University Hospital de La Princesa, CIBER-CV, IIS-IP, Universidad Autónoma de Madrid, c/ Diego de León 62, 28006 Madrid, Spain; 2 Internal Medicine Department, University Hospital Gregorio Marañon, Madrid, Spain; 3 Internal Medicine Department, University Hospital de la Princesa, Madrid, Spain; 4 Internal Medicine Department, University Hospital of Cabueñes, Gijón, Spain; 5 Radiology Department, University Hospital de la Princesa, Madrid, Spain; 6 Pharmacy Department, University Hospital of Cabueñes, Gijón, Spain; 7 Pneumology Department, University Hospital de la Princesa, Madrid, Spain; 8 Geriatric Department, University Hospital de la Princesa, Madrid, Spain; 9 Immunology Department, University Hospital de la Princesa, CIBER-CV, IIS-IP, Universidad Autónoma de Madrid, Madrid, Spain; 10 Data Analysis Department, University Hospital de la Princesa, Madrid, Spain; 11 Department of Anaesthesia and Intensive Care, IRCCS Istituto Clinico Humanitas, Humanitas University, Milan, Italy; 12 Clinical Analysis Department, University Hospital de la Princesa, Madrid, Spain

**Keywords:** Randomized controlled trials, Respiratory tract diseases, Infectious diseases, Inflammatory diseases

## Abstract

Some patients with COVID-19 pneumonia develop an associated cytokine storm syndrome that aggravates the pulmonary disease. These patients may benefit of anti-inflammatory treatment. The role of colchicine in hospitalized patients with COVID-19 pneumonia and established hyperinflammation remains unexplored. In a prospective, randomized controlled, observer-blinded endpoint, investigator-initiated trial, 240 hospitalized patients with COVID-19 pneumonia and established hyperinflammation were randomly allocated to receive oral colchicine or not. The primary efficacy outcome measure was a composite of non-invasive mechanical ventilation (CPAP or BiPAP), admission to the intensive care unit, invasive mechanical ventilation requirement or death. The composite primary outcome occurred in 19.3% of the total study population. The composite primary outcome was similar in the two arms (17% in colchicine group vs. 20.8% in the control group; *p* = 0.533) and the same applied to each of its individual components. Most patients received steroids (98%) and heparin (99%), with similar doses in both groups. In this trial, including adult patients with COVID-19 pneumonia and associated hyperinflammation, no clinical benefit was observed with short-course colchicine treatment beyond standard care regarding the combined outcome measurement of CPAP/BiPAP use, ICU admission, invasive mechanical ventilation or death (Funded by the Community of Madrid, EudraCT Number: 2020-001841-38; 26/04/2020).

## Introduction

The severe acute respiratory syndrome coronavirus-2 (SARS-CoV2) infection, responsible for coronavirus disease 2019 (COVID-19), is associated with high mortality secondary to pneumonia^1^. Current evidence suggests that some patients with COVID-19 pneumonia develop an associated cytokine storm syndrome that aggravates the pulmonary disease^2^. The use of dexamethasone^3^ has been was shown to reduce mortality in these patients, supporting the importance of the inflammatory process.

Colchicine is an anti-inflammatory drug with appealing features for the treatment of the associated hyperinflammation in this setting. First, colchicine inhibits the production of chemokines involved in leukocyte recruitment and activation^4,5^. Second, colchicine may have an immunomodulatory effect on pulmonary leukocytes^6^. Third, what in another context would be considered an adverse effect leading to a greater risk of pneumonia, in the COVID-19 hyperinflammation phase, here may act as an effective treatment. Moreover, COVID-19 has been associated with cardiac complications^7,8^, and therefore, colchicine, as a cardioprotective drug^9^, could prevent this associated damage. Finally, colchicine is characterized by a safe profile in terms of adverse effects.

The COLCORONA trial, a randomized, double-blind, placebo-controlled, investigator-initiated trial, included 4488 non hospitalized patients with SARS-CoV-2 infection; in the predefined subset of patients with PCR-confirmed COVID-19, colchicine reduced the composite rate of death or hospitalizations^10^. Colchicine in hospitalized patients with SARS-CoV-2 infection has been previously assessed. In the GRECCO-19 trial, an open-label randomized trial including 105 hospitalized patients for SARS-CoV-2 infection, colchicine improved time to clinical deterioration; however, no significant difference was observed in clinical respiratory complications or death^11^. Recently, the RECOVERY trail, a randomized, controlled, open-label trial including 11,340 hospitalized patients with COVID-19, showed no differences in the composite endpoint of invasive mechanical ventilation or death^12^.

Nevertheless, none of these studies specifically focused on the important subset of patients with associated hyperinflammation.

The role of colchicine in hospitalized patients with moderate to severe COVID-19 pneumonia and established hyperinflammation remains unknown.

The primary aim of the trial was to determine whether adding colchicine to standard of care background therapy reduces clinical events in hospitalized patients with moderate to severe COVID19 pneumonia and established hyperinflammation, compared to standard treatment (EudraCT Number: 2020-001841-38; 26/04/2020).

## Methods

We conducted a prospective, randomized controlled, observer-blinded endpoint (PROBE), investigator-initiated trial.

### Study population

Hospitalized patients with COVID-19 pneumonia were eligible if they were ≥ 18 years of age and presented with at least 2 of the following 4 inflammatory criteria: C-reactive protein > 4 mg/dL, D-dimer > 1 mg/L, ferritin > 1000 ng/mL or fever ≥ 38 °C in the last 24 h. The diagnosis of SARS-CoV-2 was made using polymerase chain reaction testing on a nasopharyngeal swab specimen in all patients. All patients had a chest X-ray test assessed by expert radiologists supporting the diagnosis of pneumonia. Furthermore, radiologists assigned each chest X-ray a RALE score, reflecting the extent of lung involvement from COVID-19 pneumonia^13^.

Patients were excluded if they had concomitant active inflammatory bowel disease, diarrhea or malabsorption; estimated glomerular filtration rate less than 30 mL/min/1.73 m^2^; severe hepatic cirrhosis or acute hepatitis; granulocytopenia (< 500/mm^3^); low platelet count (< 50 × 10^9^/L); concomitant treatment with ritonavir; chronic treatment with colchicine or immunosuppression treatment; history of significant sensitivity to colchicine; pregnant women, or participating in another clinical trial. Patients were excluded if they were already receiving either non-invasive or invasive respiratory support at the time of screening.

Clinicians assessed the initial eligibility of the patients. Thereafter, masked randomization was centralized and done electronically by the principal investigators of each site using an automated interactive web-response system (www.studyrandomizer.com). Recruited patients were randomly assigned, in a 1:1 ratio, to colchicine (5 days of oral treatment: 1 mg loading dose and then 0.5 mg/day) or no colchicine, using an allocation sequence that was computer-generated using a blocking schema with block sizes of four. Allocation sequence was not stratified.

An academic steering committee designed the trial protocol (available as Supplemental material) and oversaw the study execution. The clinical research pharmacy monitored the appropriate drug administration. Four tertiary university hospitals participated in the study. Sites and investigators are presented in the Online Appendix. For each analysis, patients with missing value were excluded following a strategy of complete-case analysis. Regarding quality control, the data of all patients were recorded electronically in a database created for this purpose. The database was debugged by means of logical, range and consistency tests. Once the data had been linked, the patient identifiers were anonymized. Each patient, or the patient’s legally authorized representative, provided informed consent. The trial was conducted in accordance with the International Conference on Harmonisation E6 guidelines for Good Clinical Practice and the Declaration of Helsinki. This trial was approved by the Spanish Agency of Medicines and Medical Devices and all the corresponding local Clinical Research Ethics Committee (Hospital Universitario de La Princesa, Hospital General Universitario Gregorio Marañon, Hospital Universitario de Cabueñes and Hospital Universitario Fundación Jiménez Díaz).

### Main outcomes

The primary efficacy outcome measure was a composite of non-invasive mechanical ventilation (CPAP or BiPAP), admission to the intensive care unit, invasive mechanical ventilation requirement or death. The primary outcome measure was overseen and adjudicated by an independent clinical event committee which was blinded to treatment allocation. In patients previously suffering from sleep apnea syndrome, the use of CPAP was only considered as an outcome if clinically indicated for oxygenation therapy after failure of standard oxygenation support. Secondary outcomes were hospital stay and changes in laboratory biomarkers of inflammation and troponin. The incidence and severity of adverse events were prospectively evaluated.

### Statistical analyses

Quantitative variables are presented as mean ± standard deviation or median (interquartile range) as required. Comparisons between both arms were made with the Student’s t-test or Mann–Whitney–Wilcoxon test. Categorical variables are expressed as frequency (percentage) and both arms were compared with χ^2^ test and Fisher’s exact test when necessary. It was estimated that a sample size of approximately 240 randomized patients, with 120 patients in each treatment arm, would be required to detect a 50% relative risk reduction with colchicine, considering a primary endpoint event rate of 30% in the control group, with a power of 80% and a two-sided test at the 0.05 significance level. The efficacy analyses were conducted using an intention-to-treat analysis. The composite primary outcome, as well as its components, were compared between the two groups using logistic regression analyses and odds ratios (95%CI) were provided. In addition, pre-specified subgroup analyses of the composite primary outcome measure were performed according to well-established risk factors for poor outcome in COVID-19, including age, sex, hypertension, body mass index (BMI), days from onset of symptoms, pneumonia extension in chest X-ray and oxygen support requirement at recruitment^3,13–17^. Statistical analyses were performed using STATA 14.2 package (StataCorp, LLC, Texas).

## Results

**Figure 1:**
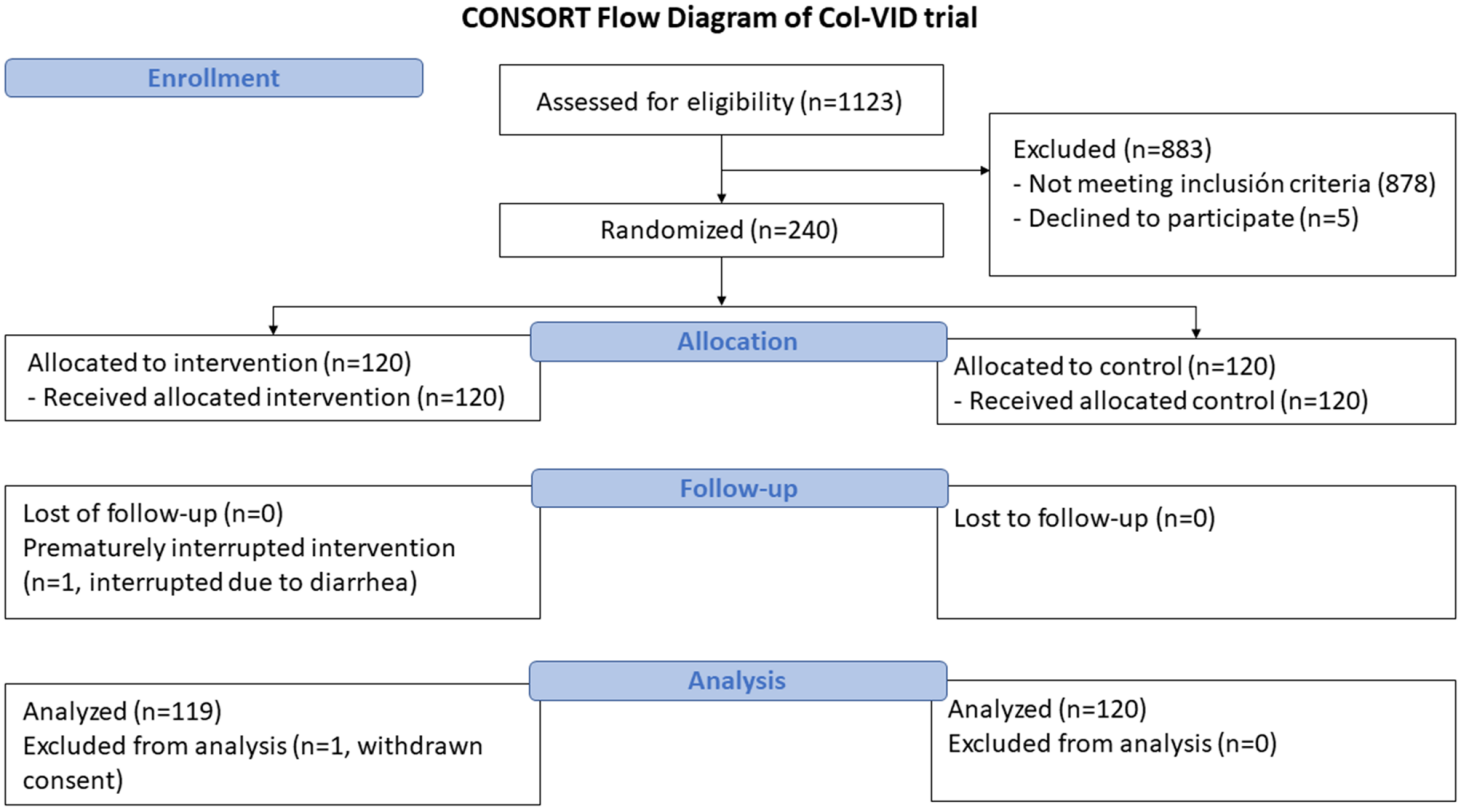
Consort flow diagram. The progress of all participants through the trial is displayed.

All patients were recruited between August 2020 and March 2021. Of them, one patient withdrew consent. Finally, 119 patients formed the colchicine group and 120 the control group (Fig. 1).

The baseline characteristics of patients are shown in Table 1. Patients were enrolled with a mean of 1.9 ± 1.9 days after hospital admission and a mean of 9.0 ± 3.4 days after the onset of symptoms. The mean age was 65 ± 16 years and 59% were male. Mean BMI was 27.5 ± 4.5, 16% had diabetes, 40% were hypertensive and 7% had a history of coronary artery disease. The two groups showed no significant differences in the clinical baseline characteristics. According to the WHO disease severity classification, 53 (22%) and 186 (78%) participants had moderate and severe COVID-19 disease, respectively. No differences were observed between the two groups on disease severity. Considering days from admission, the control group patients were recruited slightly earlier (2.1 ± 2.3 days in the colchicine group vs. 1.6 ± 1.1 days in the control group; *p* = 0.026). However, no difference was detected in terms of days from the onset of symptoms.

**Table 1:** Baseline characteristics.

	Global	Colchicine N = 119	Control N = 120	*p*
Age, years	65.1 ± 16.0	66.0 ± 15.5	64.2 ± 16.4	0.397
Sex (male)	141 (59%)	70 (59)	71 (59%)	0.957
Body mass index	27.5 ± 4.5	27.1 ± 4.1	27.8 ± 4.7	0.255
Hypertension	95 (40%)	49 (41%)	46 (38%)	0.653
Dyslipidemia	78 (33%)	42 (35%)	36 (30%)	0.393
Diabetes mellitus	39 (16%)	20 (17%)	19 (16%)	0.839
Smoker	10 (4%)	6 (5%)	4 (3%)	0.509
Coronary artery disease	16 (7%)	9(8%)	7 (6%)	0.593
Heart failure	6 (3%)	5 (4%)	1 (1%)	0.096
Sleep Apnea Syndrome	6 (3%)	1 (1%)	5 (4%)	0.100
Asma	12 (5)	4 (3%)	8 (7%)	0.242
Chronic obstructive pulmonary disease	10 (4%)	4 (3%)	6 (5%)	0.527
Days from the onset of symptoms	9.0 ± 3.4	9.0 ± 3.4	9.0 ± 3.4	0.954
Days from admission	1.9 ± 1.9	2.1 ± 2.3	1.6 ± 1.1	0.026
FiO_2_ for SatO_2_ > 90%	0.30 ± 0.11	0.30 ± 0.11	0.30 ± 0.12	0.911
Temperature, °C	37.3 ± 1.0	37.2 ± 1.0	37.4 ± 1.0	0.347
C-reactive protein, mg/dL	9.8 ± 7.6	9.2 ± 6.2	10.4 ± 8.8	0.230
Ferritin, ng/mL	1203.5 ± 929.2	1346.9 ± 1023.6	1053.6 ± 796.3	0.018
D-dimer, mg/L	1.0 ± 0.8	0.9 ± 0.7	1.1 ± 0.9	0.026
Interleukin-6	40.1 ± 101.4	36.9 ± 62.9	43.6 ± 131.1	0.635
hs-TnT, ng/L	11.2 ± 16.4	11.8 ± 21.0	10.6 ± 9.7	0.611

*hs-TnT* high sensitive Troponin T.

Regarding inflammation markers at recruitment, mean ferritin was higher in colchicine group (1346.9 ± 1023.6 ng/mL in colchicine group vs. 1053.6 ± 796.3 ng/mL in control group; *p* = 0.018) while mean d-dimer was higher in control group (0.9 ± 0.7 mg/L in colchicine group vs. 1.1 ± 0.9 mg/L in control group; *p* = 0.026) mg/L. No significant differences were observed in temperature, C-reactive protein and interleukin 6 values.

### Clinical endpoints

The composite primary outcome occurred in 19.3% of the total study population. Specifically, 10.5% were admitted to intensive care units, 14.2% received non-invasive ventilation (CPAP or BiPAP), 6.3% were invasively ventilated and 6.7% died during hospitalization. The composite primary outcome was similar in the two arms (17% in the colchicine group vs. 20.8% in the control group; *p* = 0.533) and the same applied to each of its individual components (Table 2).

**Table 2:** Composite primary endpoint and individual components.

	Colchicine (n = 119)	Control (n = 120)	OR	95% CI	*p*
Composite primary endpoint	21 (17.7%)	25 (20.8%)	0.81	0.43–1.55	0.533
CPAP/BiPAP	16 (13.5%)	18 (15.0%)	0.88	0.43–1.82	0.731
ICU admission	11 (9.2%)	14 (11.7%)	0.77	0.33–1.78	0.541
Invasive ventilation	5 (4.2%)	10 (8.3%)	0.48	0.16–1.46	0.196
Death	7 (5.9%)	10 (8.33%)	0.69	0.25–1.87	0.463

*CPAP/BiPAP* continuous positive airway pressure/bilevel positive airway pressure, *ICU* intensive care unit.

Most patients received steroids (98%) with a similar peak dose of steroids in both groups (17.9 ± 12.1 mg of dexamethasone equivalent dose in colchicine group and 16.6 ± 12.8 mg in the control group; *p* = 0.418). A 99% of the patients received heparin (59.9% prophylactic, 31.4% intermediate and 8.0% anticoagulant doses) with no difference between the groups. None of the patients received hydroxychloroquine. Two patients of the colchicine group received lopinavir/ritonavir. Remdesivir was administered in 37 patients (19 patients in the colchicine group vs. 18 in the control group; *p* = 0.859). A total of 47 patients received tocilizumab during the hospitalization (30 in the colchicine group vs. 17 in the control group; *p* = 0.035). The use of other monoclonal antibodies was very minimal (anakinra to 1 patient in each group; baricitinib was administered to 1 patient in colchicine group and to 2 patients in the control group).

**Figure 2:**
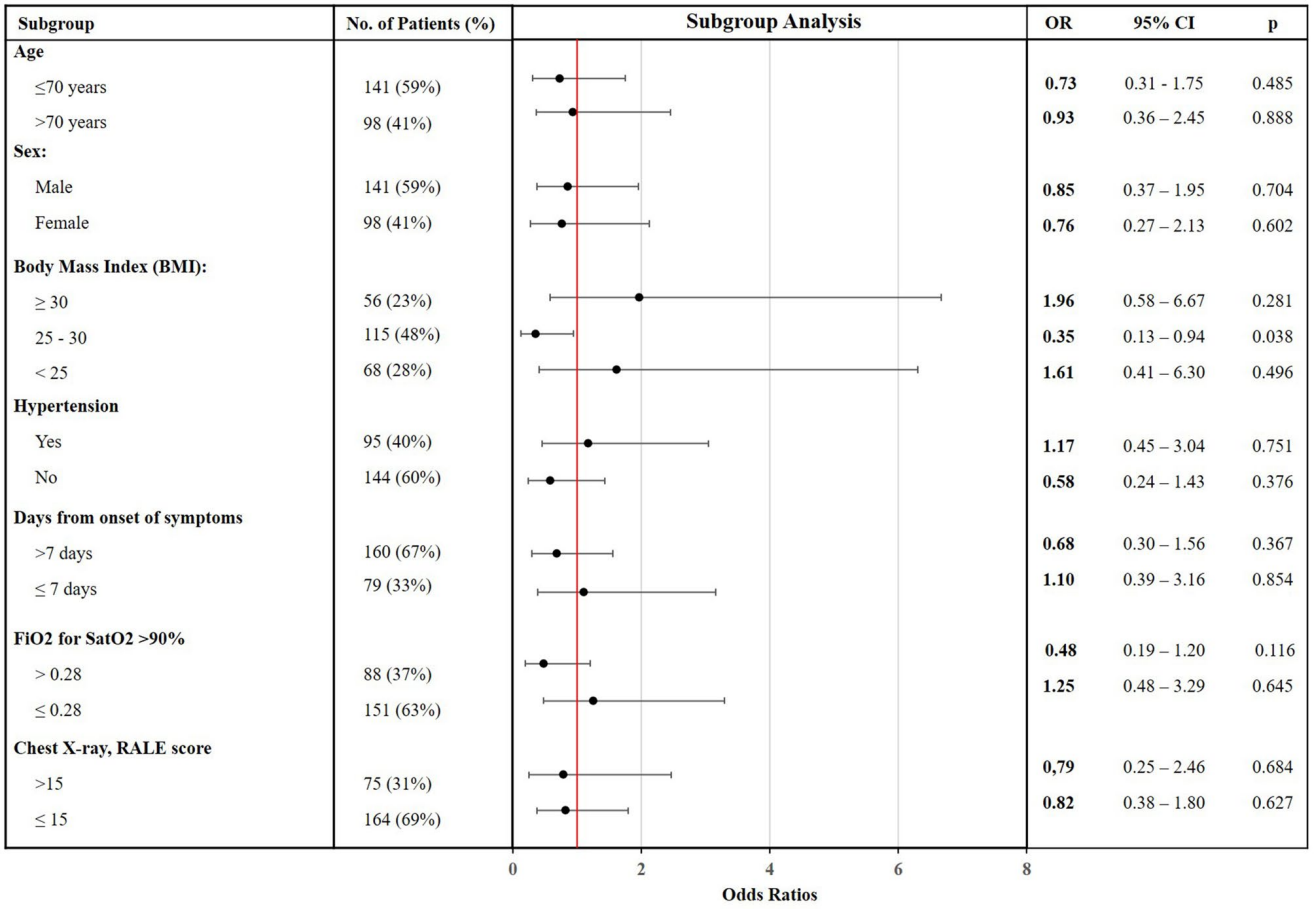
Forest plot for the effect of colchicine on the composite outcome in prespecified subgroups. *OR* odds ratios, *95% CI* 95% confidence interval.

Pre-specified subgroup analyses are shown in Fig. 2. Results of most subgroups were consistent with the main analysis. However, in overweight patients, the colchicine group showed a lower prevalence of the composite primary outcome (11.7% in the colchicine group vs. 27.3% in the control group; OR 0.35, 95% CI 0.13–0.94; *p* = 0.038).

Mean hospital stay was 11.3 ± 9.6 days. No differences were seen between the two groups (*p* = 0.765).

### Inflammatory laboratory markers and troponin

**Figure 3:**
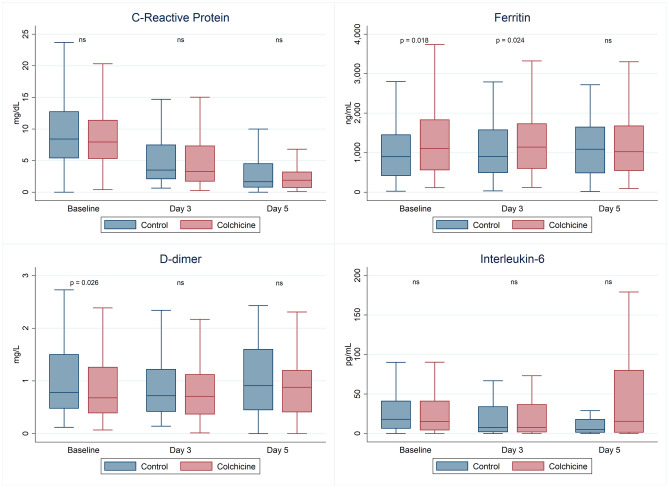
Inflammatory markers. Distribution of inflammatory markers in each group and over time are presented using box-and-whisker plots. Significant comparisons are shown.

Results of inflammatory markers at recruitment, on the 3rd and 5th day are shown in Fig. 3. Apart from the difference at recruitment commented above, no statistically significant differences were found on the inflammatory biomarkers on the 3rd and 5th day, except for the ferritin on the 3rd day in which higher levels for colchicine were found (*p* = 0.024). Troponin showed no differences both at recruitment (12 ± 21 ng/L in the colchicine group vs. 11 ± 10 ng/L; *p* = 0.661) and at 5th day (15 ± 33 ng/L in the colchicine group vs. 9 ± 8 ng/L; *p* = 0.101).

### Adverse events

Following exclusion criteria, none of the patients had diarrhea at recruitment. Diarrhea incidence was infrequent and similar in both groups (3.4% in the colchicine group vs. 1.7% in the control group; *p* = 0.683). Colchicine was interrupted prematurely due to diarrhea only in one patient. Thrombotic events, defined as deep vein thrombosis or pulmonary embolism, were diagnosed in 1.7% of patients in the colchicine group and 4.2% of those in the control group (*p* = 0.446). No hematologic disorders or abnormalities were detected.

## Discussion

To our knowledge, this is the first study assessing the clinical efficacy of colchicine in hospitalized patients with COVID-19 pneumonia and established hyperinflammation. In this randomized trial adding colchicine to standard background therapy was not associated with a lower risk of CPAP/BiPAP use, ICU admission, invasive ventilation requirement or death.

When the study was designed, COVID-19 patients had worse clinical outcomes at the beginning of the pandemic as steroids and heparins had not yet been included in the standard treatment protocols. Nonetheless, most patients included in our trial received steroids and heparins, potentially explaining, at least in part, a lower than expected rate of the observed primary clinical endpoint. For this reason, the main analysis might have been underpowered. Moreover, the optimal dosage of colchicine in COVID-19 patients is still undefined. Remarkably, the proposed dose of colchicine was safe in our population with moderate to severe COVID-19 pneumonia.

The SARS-CoV-2 interaction with macrophages promotes the nod-like receptor protein 3 (NLRP3) inflammasome assembly, a proinflammatory complex which drives to several cytokines release including IL-1β and IL-6. These pro-inflammatory cytokines induce neutrophil activation and infiltration into the infected tissues, which, in excess, aggravate the clinical condition. Considering that colchicine has shown to inhibit NLRP3 inflammasome and neutrophil activation, it has been considered a valid candidate as potential treatment for COVID-19^18,19^. Two randomized clinical studies previously suggested a beneficial effect of colchicine in hospitalized patients with COVID-19, recruiting only 72 and 105 patients, respectively. Specifically, Lopes et al. observed a reduction of hospital stay, whereas Deftereos et al. documented a significant improvement in time to clinical deterioration in those participants receiving colchicine^11,20^. However, the results remained uncertain as both trials had a small sample size and a low rate of events. On the other hand, the colchicine arm of the RECOVERY trial, a large randomized clinical trial, found no clinical benefit. Moreover, colchicine showed no beneficial effect on the need of mechanical ventilation or 28-day mortality in a recent randomized clinical trial including 1279 patients hospitalized with COVID-19 pneumonia^21^. Nevertheless, none of these previous studies focused on patients with COVID-19 pneumonia and established hyperinflammation, a subset of patients particularly attractive to assess the effect of colchicine.

In the pre-specified subgroup analysis, overweight patients in the colchicine group showed a statistically significant lower prevalence of CPAP/BiPAP use, ICU admission, invasive ventilation or death. Interestingly, in the scenario of SARS-CoV-2 infection, overweight and obesity are associated with a higher risk of hospital admission^22^ and progression to severe COVID^23^. However, since the RECOVERY and ECLA PHRI COLCOVID trials did not collect BMI information^12,21^, this is the first clinical trial analyzing the effect of colchicine on overweight patients. Remarkably, the excess of macronutrients in the adipose tissues predisposes to the releases of inflammatory mediators which participate in the cytokine storm aggravating COVID-19^2^. Furthermore, colchicine was previously demonstrated to significantly improve the inflammatory markers in obese patients without significant medical illness^24^. For all these reasons, overweight patients with COVID-19 pneumonia and established hyperinflammation might particularly benefit from colchicine treatment. In a post-hoc analysis we found no differences in the background therapy of overweight patients during hospitalization, including tocilizumab. On the other hand, proposed dose and treatment duration of colchine might be insufficient to observe beneficial effects on obese patients. Thus, despite being appealing from a pathophysiological standpoint, major care should be paid to interpret this subgroup considering its limited size. Therefore, results should be only considered as hypothesis-generating and would require confirmation in further studies.

Colchicine had no beneficial effect on myocardial injury measured as troponin release on the 5th day. However, considering the stabilizing effect of colchicine on atherosclerotic plaques^5,9^, patients with coronary artery disease would theoretically be the subgroup of patients more responsive to colchicine. Since coronary artery disease was present only in 6.7% of the study patients, our cohort may not be appropriate to rule out a potentially protective role of colchicine on myocardial injury associated with COVID-19.

Our study has several limitations. As discussed, the event rate was lower than expected, therefore, reducing the power of the main analysis. Besides, the lack of placebo may introduce a performance bias that cannot be excluded. However, this was partially mitigated by the blinding of outcome assessment using an independent external clinical event committee. Despite the short-course colchicine treatment, a longer biological effect was expected as the drug’s half-life is of about 60 h in leukocytes. Despite using a masked randomization scheme, which is a common method to mitigate allocation biases in baseline characteristics, our study showed differences between groups in term of ferritin and d-dimer levels at randomization. These differences in inflammatory markers might explain the higher rate of tocilizumab administration in the colchicine group during the hospital stay.

## Conclusions

In a cohort of 240 hospitalized adult patients with COVID 19 pneumonia and associated hyperinflammation, no clinical benefit was observed with short-course colchicine treatment beyond standard care regarding the combined outcome measurement of CPAP/BiPAP use, ICU admission, invasive mechanical ventilation or death. Further studies are warranted to identify COVID-19 patients potentially experiencing a clinical benefit with colchicine treatment.

## Supplementary Information

Supplementary Information.

## Data Availability

Data sharing will be considered upon reasonable request including a detailed research plan with the corresponding author.
