# Supplementary material for: Efficacy of short-course colchicine treatment in hospitalized patients with moderate to severe COVID-19 pneumonia and hyperinflammation: a randomized clinical trial
Source: Sci Rep. 2022 Jun 2;12:9208. doi: 10.1038/s41598-022-13424-6 (PMC9161184; doi:10.1038/s41598-022-13424-6)
Supplement: Supplementary file 1 — Supplementary Information. [file 41598_2022_13424_MOESM1_ESM.docx]

**ONLINE APPENDIX.**

**Sites and Investigators:**

1 Hospital Universitario de La Princesa, CIBERCV, Madrid, Spain (Alberto Cecconi, Pablo Martínez Vives, Alberto Vera Sainz, Ana Barrios, Diana Prada, Susana Hernández Muñiz, María José Olivera, Marianela Ciudad, Azucena Bautista, Jesus Sanz, Elena García Castillo, Gorane Iturricastillo, Elena Ávalos, Carolina Núñez de Arenas Liberos, Natalia Fernanda Pascual Gomez, Pilar Hernández, Paloma Gil, Maria Jesus Delgado, Lucio García Fraile, Nuria Ruiz- Giménez, Jose Virgilio Torres, Enrique de la Vega, Nicolás Labrador, Pedro Parra, Pablo Rodríguez, Beatriz Sánchez, José María Galván, Ancor Sanz García, Guillermo Ortega, María Varela Piñón, Ángela Gutiérrez, Tamara Alonso, Celeste Marcos, Inés Ruiz, Elisa García Besteiro, Hortensia de La Fuente, Arantzazu Alfranca, Francisco Abad Santos, Paloma Caballero, Maria Angeles Sanz de Benito, Francisco Sanchez-Madrid , Luis Jesus Jimenez-Borreguero, Julio Ancochea, Carmen Suarez, Fernando Alfonso)

2 Hospital Universitario Gregorio Marañon, Spain (Cristina Lavilla, Alvaro Alejandre de Oña, Eduardo Fernandez Carracedo)

3 Hospital Universitario de Cabueñes, Gijón, Spain (Eva Fonseca, Rubén Pampín Sánchez, José Miguel Vegas Valle, Maria Rosa Fernández-Madera Martínez, Cristina Helguera Amezua, María Luisa Taboada Martínez)

4 Hospital Universitario Fundación Jimenez-Diaz, Spain (Felipe Villar)

^5^IRCCS Istituto Clinico Humanitas, Humanitas University, Milan, Italy (Maurizio Cecconi)

**Sponsor:** Spanish Society of Cardiology

**Data and Safety Monitoring Board:** A. Cecconi, LJ Jimenez-Borreguero, F. Alfonso

**Clinical Events Committee**: Juan Sanchis (Servicio de Cardiología. Hospital Clínico Universitario de Valencia. Universidad de Valencia. CIBERCV) and Borja Ibanez (Director Clinical Research CNIC & Interventional Cardiologist FJD)
